# Association between 35 blood and urine biomarkers and oral leukoplakia: a two-sample Mendelian randomization study

**DOI:** 10.3389/fonc.2024.1437493

**Published:** 2024-08-22

**Authors:** Yu-long Ouyang, Jing Wei, Mei-yan Liu, Yi Zhang, Sheng-hui Liu, Hong-chao Feng

**Affiliations:** ^1^ College of Stomatology, Guizhou Medical University, Guiyang, China; ^2^ Department of Oral and Maxillofacial Surgery, Guiyang Hospital of Stomatology, Guiyang, China; ^3^ School of Anesthesiology, Guichou Medical Unirersity, Guiyang, China

**Keywords:** Mendelian randomization study, biomarker in blood and urine, oral leukoplakia, dyslipidemia, oral potentially malignant disorders, Apolipoprotein B, non-albuminous proteinuria

## Abstract

**Background:**

Several existing studies have shown a correlation between some of the blood and urine biomarkers and oral leukoplakia (OLK). However, the causality of this relationship remains uncertain. Thus, this study aimed to examine the causal association between 35 blood and urine biomarkers and OLK.

**Methods:**

Single nucleotide polymorphisms (SNPs) associated with 35 blood and urine biomarkers were selected as instrumental variables (IVs) using a two-sample Mendelian randomization(MR) study to assess the causal relationship between the biomarkers and the risk of oral leukoplakia. We used the inverse variance weighted (IVW) method as the main analysis. Furthermore, several sensitivity analyses were performed to assess heterogeneity, horizontal pleiotropy, and stability.

**Results:**

Based on the selection criteria of the Inverse Variance Weighted (IVW) method, the analysis found that 5 blood and urine biomarkers were significantly associated with the development of leukoplakia, of which the results of IVW showed that abnormalities of Apolipoprotein B (Apo B), Cholesterol, Low-density Lipoprotein (LDL), Triglycerides (TG) promoted the development of oral leukoplakia, and Non Albumin Protein (NAP) had a protective effect on the development of oral leukoplakia. We then performed a Bonferroni correction for these results, and after correction Apo B was still causally associated with the development of oral leukoplakia (IVW P<0.0007), whereas the other four biomarkers could only provide some evidence of predisposition.

**Conclusion:**

Our two-sample Mendelian randomization study supports the existence of a causal relationship between these five blood and urine biomarkers and the occurrence of oral leukoplakia, and provides evidence for a number of risk and protective factors for the development of oral leukoplakia; however, the definitive mechanisms for the occurrence and development of oral leukoplakia still remain to be elucidated, and further studies on these relevant mechanisms are still needed.

## 1 Introduction

Oral leukoplakia refers to patchy damage of white or grayish-white keratotic lesions occurring only on the oral mucosa, of which WHO defines it as ‘A predominantly white lesion of the oral mucosa that cannot be characterized by any other definable lesion’ (1), and it is a common non-infectious, chronic disease, which can occur in all the mucous membranes of the oral cavity but is most frequent in the buccal and lingual areas, and is an Oral potentially malignant disorders (OPMDs) (2). In addition to white color, white spots can also show red and white damage. The histopathologic changes are characteristic of precancerous damage: epithelial dysplasia (3). Estimates of the global prevalence of oral leukoplakia range from 0.5% to 3.4%, with patients being predominantly middle-aged and older men (4). Some studies have shown some correlation between dyslipidemia and β2-microglobulin (5). However, traditional observational studies are susceptible to bias due to reverse causation and are unable to elucidate the causal relationship. Mendelian randomization, which uses genetic variation as an instrumental variable, can be used to assess causality between exposures and outcomes (6), and is able to overcome the bias caused by confounding and the problem of reverse causality. In this study, the exposure file and the outcome file of the two-sample Mendelian randomization came from 2 different cohorts.

## 2 Materials and methods

### 2.1 Data sources

The data for the 35 blood and urine biomarkers were derived from a genetic study of blood and urine biomarkers. In this study, researchers analyzed a large number of genetic variants, and their results describe the genetic basis of biomarkers in blood and urine, their causal impact on disease, and improved genetic risk stratification for common diseases (7). GWAS summary statistics for oral leukoplakia were obtained from the FinnGen (9th release). The GWAS for ‘oral leukoplakia’ used in this study included 376,803 individuals, including 474 cases and 376,329 controls.

### 2.2 Instrumental variables

IVs in Mendelian randomization studies must satisfy three core assumptions: (i) the assumption of relevance: the instrumental variable is strongly correlated with the exposure, with an F-value >10 as the criterion for a strong correlation; (ii) the assumption of exclusivity: the instrumental variable does not affect the outcome. (iii) Independence assumption: the instrumental variable is not related to other confounders (8, 9).

The criteria for selecting IVs were as follows: (1) a threshold of significance (P<5.0×10-6) for single nucleotide polymorphisms (SNPs) associated with each biomarker within the locus range was selected as potential IVs; (2) the linkage disequilibrium (LD) between SNPs was calculated, and among the SNPs with R^2^ <0.001 (Clumping window size=10,000kb), only the SNPs with the lowest P-value were retained; (3) SNPs with minimum allele frequency (MAF) ≤0.01 were excluded.

### 2.3 Statistical analysis

Data analysis in this study was performed using R (version 4.3.3) via the TwoSample MR (0.5.6) package and MRPRESSO (1.0). MR analyses were performed using five methods: random effects Inverse Variance Weighted (IVW) (10), weighted median method (11), MR-Egger regression analysis (12), Simple mode, Weighted mode (13) to verify whether there was a causal relationship between exposure to these biomarkers and outcome oral leukoplakia. In addition, Cochran’s IVW Q was used to quantify the heterogeneity of IVs (14), and a Bonferroni correction was performed (15), while we performed a ‘Leave-One-Out’ analysis by omitting each instrumental variable SNP in turn (16). In order to assess the causal relationship between the 35 biomarkers and oral leukoplakia, we also performed a reverse Mendelian randomization analysis on the biomarkers that were found to be causally associated with oral leukoplakia in the forward Mendelian randomization analysis.

## 3 Results

**Figure 1:**
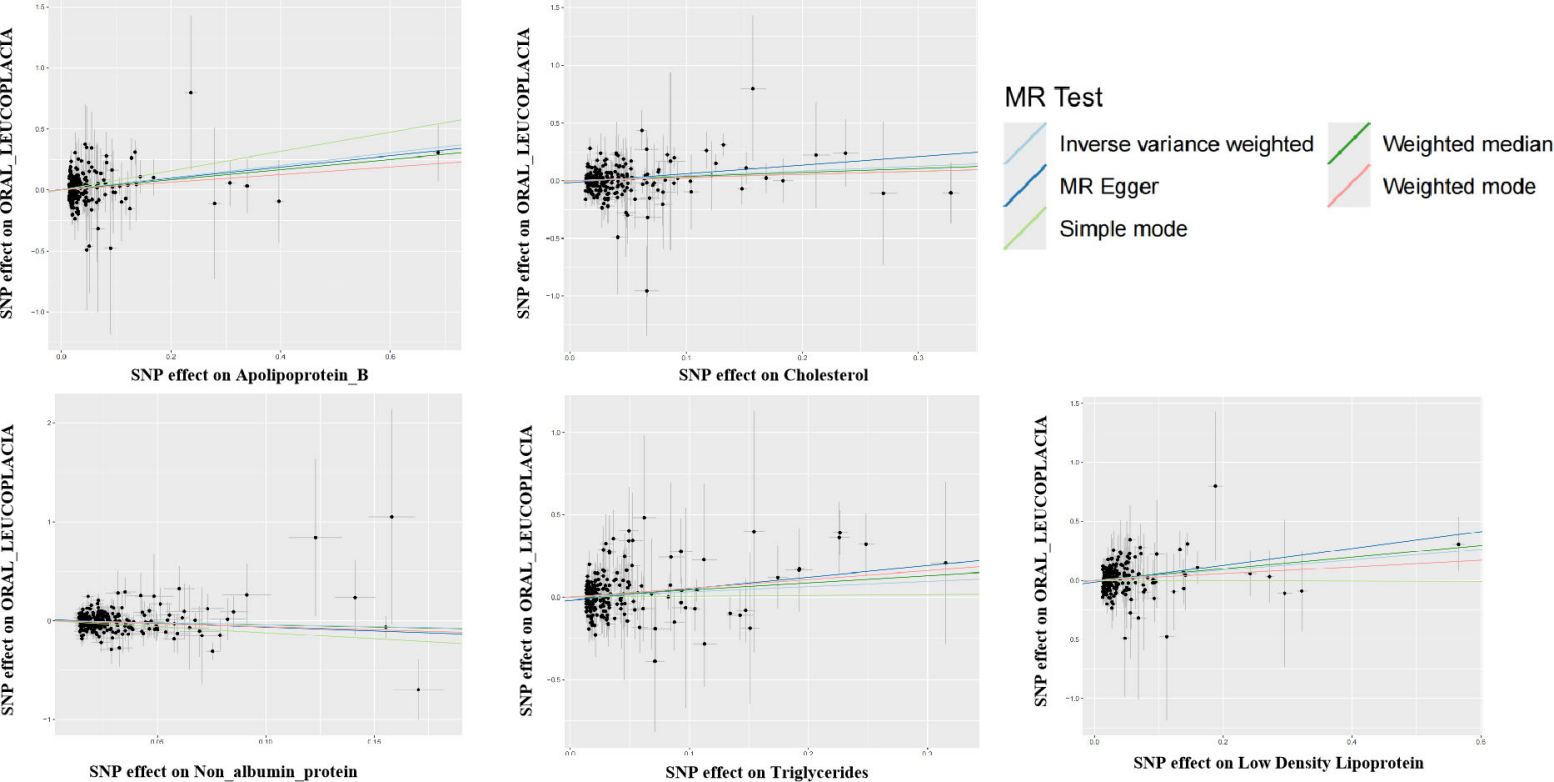
Scatter plot for the two-sample Mendelian randomization. Scatterplot of these blood vs. urine biomarker correlations with oral leukoplakia. Where each black dot represents a SNP, plotted with the correlation of SNP on exposure as the X-axis and the correlation of SNP on outcome as the Y-axis, the slope of each line marks the potential causal association of each method. The graph shows that they are causally associated with promoting the development of oral leukoplakia.

**Figure 2:**
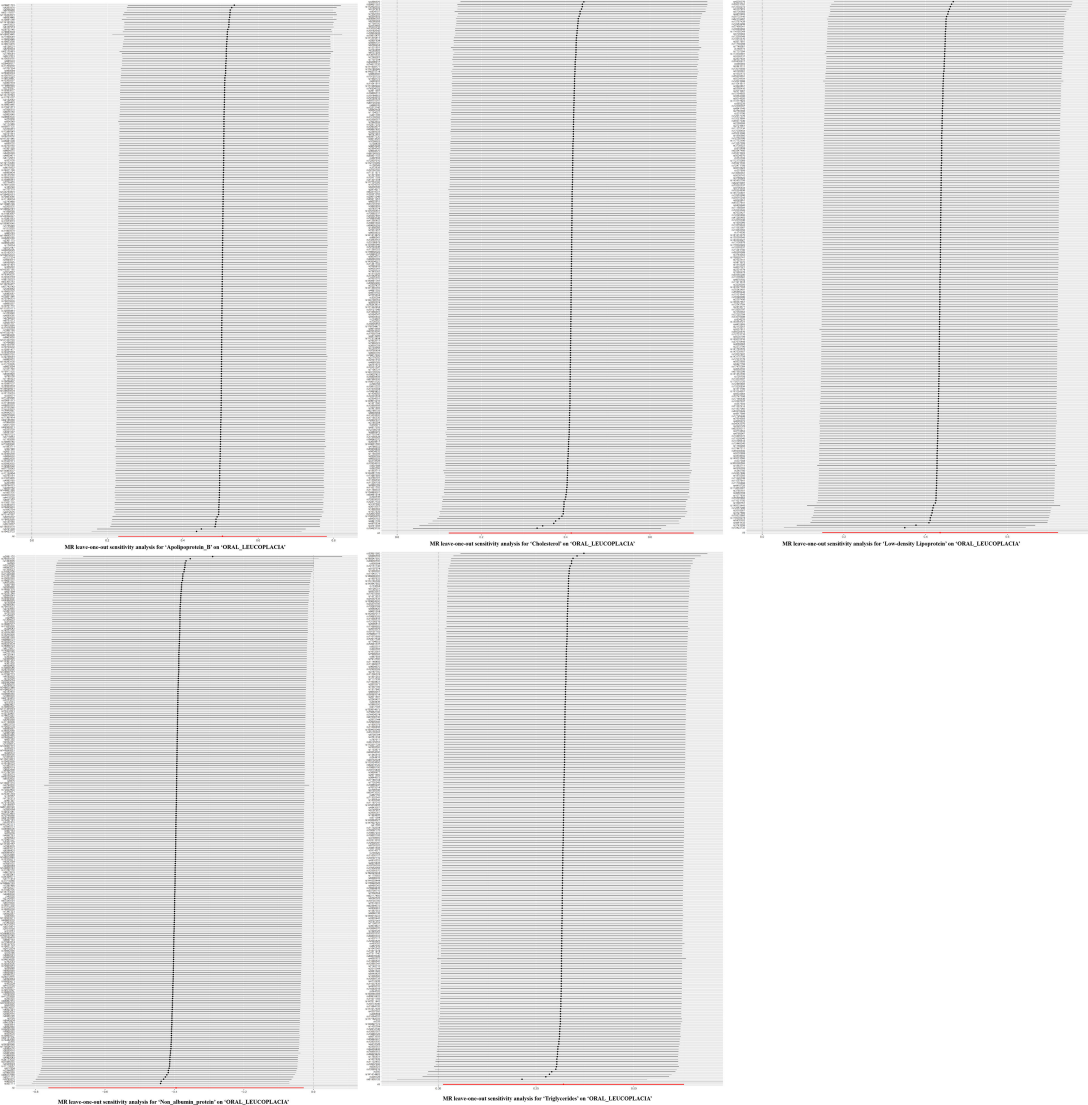
Plots of leave-one-out analyses for the two-sample Mendelian randomization analyses. Leave-one-out analyses showed no significant differences in the causal relationship between these five blood and urine biomarkers and oral leukoplakia, and after removing each SNP as an instrumental variable one by one, there was no significant change in the overall trend, and there were no SNPs in the IVs that had a large effect on the outcome.

**Figure 3:**
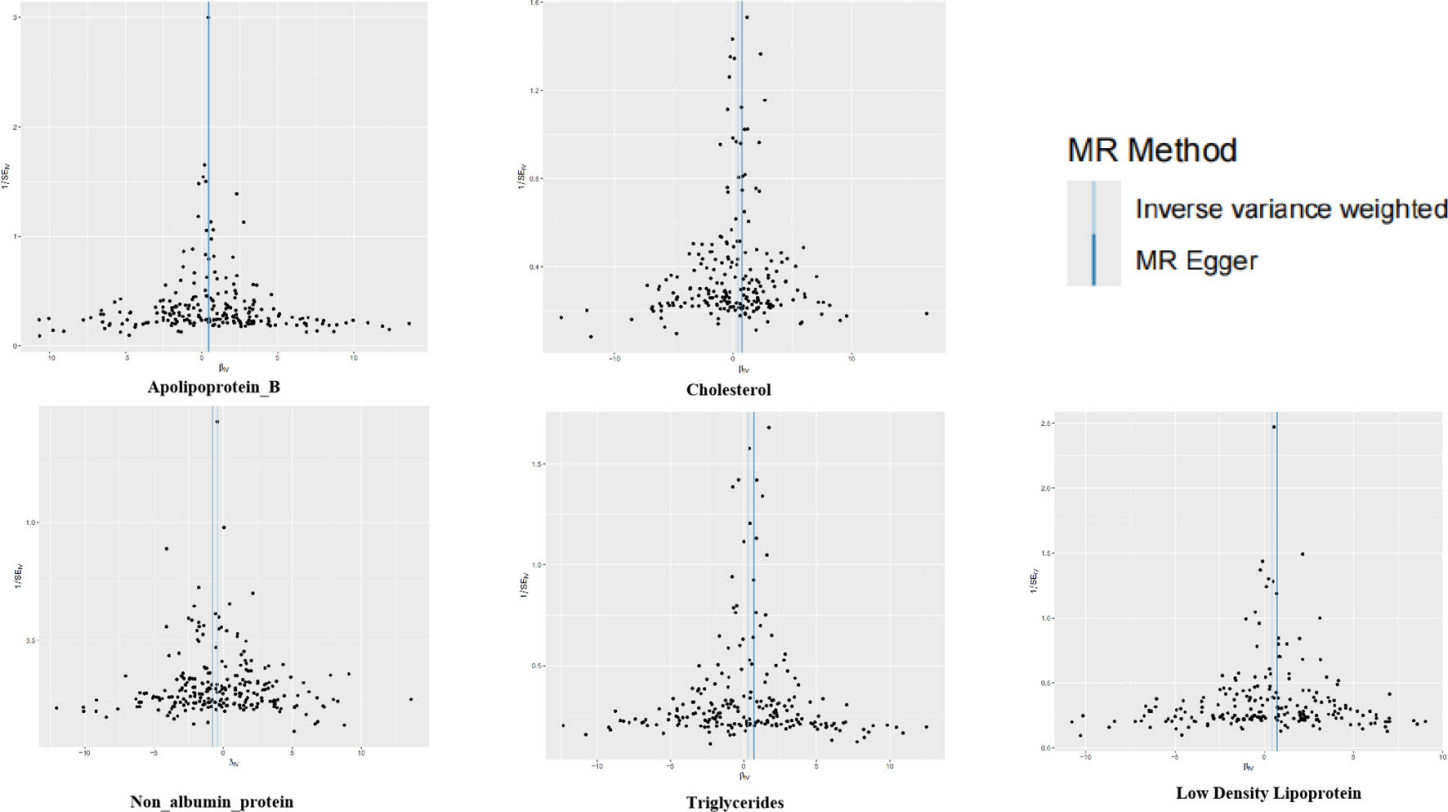
Funnel plots for the two-sample Mendelian randomization analyses. The symmetry data of the funnel plot exhibited no significant heterogeneity.

The SNPS were screened according to the selection criteria of IVs and analyzed by MR as shown in **Table 1**, five biomarkers, five blood and urine biomarkers were found to be significantly associated with the development of leukoplakia in a variety of MR methods, among which the results of IVW showed that this Apolipoprotein B, Cholesterol and Low-density Lipoprotein, Triglycerides contribute to the development of oral leukoplakia, and Non-Albumin-Protein protects against the development of oral leukoplakia. IVW results of four of these biomarkers were significantly associated with the development of oral leukoplakia. biomarkers had a promotional effect on oral leukoplakia: Apolipoprotein B (OR = 1.66, 95% CI: 1.29-2.19, P= 0.00034), Cholesterol (OR = 1.51, 95% CI: 1.13-2.02, P=0.00530), Low-density Lipoprotein, LDL (OR = 1.55, 95% CI: 1.16-2.06, P= 0.00308), Triglycerides (OR = 1.38, 95% CI: 1.01-1.87, P= 0.04158), and one of the biomarkers played a protective role against oral leukoplakia: Non Albumin Protein (OR = 0.67, 95% CI: 0.47-0.97, P= 0.03504). Scatterplots, funnel plots, and forest plots were also developed for these analyses (**Figures 1**–**3**).

**Table 1:** MR analysis values of the relationship between 35 biomarkers of blood and urine and oral leukoplakia.

Biomarker	MR Analysis method	No. of SNPs	OR	95% CI	P-value
Apolipoprotein B	MR Egger	218	1.59	1.08-2.34	0.02044
	Weighted median	218	1.52	0.92-2.51	0.10494
	**Inverse variance weighted**	**218**	**1.66**	**1.29-2.19**	**0.00034**
	Simple mode	218	2.20	0.84-5.81	0.11164
	Weighted mode	218	1.37	0.93-2.03	0.11541
Cholesterol	MR Egger	220	2.12	1.33-3.38	0.00181
	Weighted median	220	1.42	0.88-2.27	0.14755
	**Inverse variance weighted**	**220**	**1.51**	**1.13-2.02**	**0.00530**
	Simple mode	220	1.30	0.48-3.57	0.60629
	Weighted mode	220	1.30	0.77-2.21	0.32451
Low-density Lipoprotein	MR Egger	213	2.04	1.33-3.12	0.00122
	Weighted median	213	1.63	0.99-2.69	0.05461
	**Inverse variance weighted**	**213**	**1.55**	**1.16-2.06**	**0.00308**
	Simple mode	213	0.99	0.36-2.70	0.98152
	Weighted mode	213	1.33	0.83-2.14	0.23887
Non-albumin protein	MR Egger	243	0.47	0.21-1.04	0.06239
	Weighted median	243	0.66	0.34-1.27	0.21217
	**Inverse variance weighted**	**243**	**0.67**	**0.47-0.97**	**0.03504**
	Simple mode	243	0.30	0.06-1.45	0.13493
	Weighted mode	243	0.55	0.21-1.39	0.20706
Triglycerides	MR Egger	210	2.02	1.27-3.20	0.00315
	Weighted median	210	1.55	0.91-2.64	0.10944
	**Inverse variance weighted**	**210**	**1.38**	**1.01-1.87**	**0.04158**
	Simple mode	210	1.05	0.38-2.88	0.92261
	Weighted mode	210	1.71	1.06-2.78	0.02981

The bold values in the table represent the p-value for IVW, which plays a decisive role in screening positive results. (Exposure is considered to be causally related to outcome when the p-value is less than 0.05).

After Bonferroni correction the protective effect of Apolipoprotein B remained significant Apolipoprotein B [OR = 1.66, 95% CI: 1.29-2.19, P= 0.00034 (P_Bonferroni_<0.0007)], however the remaining 5 biomarkers when tested by IVW suggested correlations with oral leukoplakia, but after Bonferroni correction these correlations were no longer significant, but still suggested some predisposition for oral leukoplakia (0.05<P<0.0007). In addition the data from the reverse Mendelian randomization analysis showed that the biomarkers screened in the forward Mendelian randomization analysis that had a correlation with oral leukoplakia were not found to have a significant correlation between oral leukoplakia and the screened biomarkers in the reverse Mendelian randomization analysis (**Supplementary File**: table. Reverse MR).

In all five causal associations, the F-statistic range of the IVs was more than 10, excluding weak instrumental variables, and the Cochran ‘s IVW Q-test reflected the absence of heterogeneity in these IVs (**Supplementary File**: table. heterogeneity). In addition, to identify potentially heterogeneous SNPs, we performed a ‘Leave-one-out’ analysis by omitting each SNP in turn. Bonferroni correction was applied, and values with a p-value <0.0007 were considered to be statistically significant even after the test of multiplicity, although even if the p-value was >0.0007, some suggestive associations between these biomarkers and oral leukoplakia were still considered at p<0.05. In addition, there was no significant horizontal pleiotropy (p>0.05),as well as further MR-PRESSO analysis did not reveal any significant outliers (p>0.05) according to the results of MR-Egger regression analysis of scatterplot intercepts (**Supplementary File**: Table.res_presso), and the remaining details are shown in **Table 1**.

## 4 Discussion

We performed a two-sample Mendelian randomization analysis of 35 blood and urine biomarkers and oral leukoplakia genetic variation data to further assess the causal relationship between these 35 blood and urine biomarkers and oral leukoplakia. As a result, we found that changes in four of these biomarkers had a promoting effect: Apolipoprotein B, Cholesterol, Low-density Lipoprotein, Triglycerides, and a protective effect of Non-albumin protein. After Bonferroni correction for the five biomarkers mentioned above, it was found that there was still a causal relationship between Apolipoprotein B and oral leukoplakia, whereas the correlation between the remaining four biomarkers and oral leukoplakia was no longer significant, but there was still a suggestion of some predisposition to the development of oral leukoplakia (0.05<P<0.0007).

Several observational and controlled studies have found that oral squamous cell carcinoma and oral potentially malignant diseases (OPMDs) are associated with dyslipidemia (17), and controlled studies have found that cholesterol, LDL, and Apo B levels are significantly lower in oral cancers and patients (18). Lipids exist as membrane components of cells and are critical for a variety of biological functions, including cell division and growth of normal and malignant tissues (19). Some neoplastic or tumor cells require many essential components well above normal limits to maintain the structural and functional integrity of all biological membranes (20). Increased lipid requirements to meet the needs of these proliferating cells are expected to reduce existing lipid stores (hypocholesterolemia), low levels of lipids may be due to rapid cell division during neomembrane biogenesis as precancerous cells and malignant tumors utilize them (21, 22), and changes in these biomarkers in addition to Apo B may be risk factors for leukoplakia or suggestive of oral cancer development, and some study found elevated serum β2-microglobulin levels in oral cancer and potentially malignant diseases of the oral cavity, possibly (23, 24). Kark et al. found that cancer risk was negatively correlated with concentrations of retinol proteins and serum cholesterol, and that retinol proteins had a stronger association with cancer than serum cholesterol (25). Positive indicators in non-albumin proteins are often low molecular weight proteins: β2-microglobulin (26), α1-microglobulin, and retinol-binding proteins (27, 28), which, in combination with the above studies, suggests that non-albumin proteuric changes in the urine tests in the current Mendelian randomization may have a protective effect on the development of oral leukoplakia.

In this study, the causal relationship between biomarker in blood and urine and oral leukoplakia was determined by using Mendelian randomization analyses in 35 to exclude confounding by confounding factors and the effect of causal inversion on causal inference. The strength of IVs in Mendelian randomization analysis was ensured by parameter settings. Tests for detection and exclusion of horizontal polytropy were performed using the MR-PRESSO and MR-Egger regression intercept terms. In addition, the use of Bonferroni correction lowered the rate of false positives. There are also some shortcomings, firstly, this study used summary statistics rather than raw data in the analysis process, in this study, it can only reflect the promotional effect of Apo B on oral leukoplakia, and suggests a suggestive promotional effect of cholesterol, triglycerides, and low-density cholesterol on the development of oral leukoplakia, and a suggestive protective effect of non-albumin proteinuria on the development of oral leukoplakia However, the mechanisms involved in how they contribute to the development of oral leukoplakia have not been further explored.

## 5 Conclusion

In conclusion, in the present two-sample bidirectional Mendelian randomization study, five of the 35 blood and urine biomarkers were found to be causally associated with leukoplakia, and no correlation was suggested by these screened biomarkers in the reverse Mendelian randomization analysis. In our forward Mendelian randomization analysis, ApoB was found to contribute to the development of oral leukoplakia in 35 biomarkers of blood and urine even after Bonferroni correction (IVW: P<0.0007). The other four biomarkers suggested that changes in serum lipid profile may be a marker of development of oral leukoplakia, a potential risk factor, and an indicator for potential assessment of prognosis. Changes in non-albumin proteinuria may be suggestive of a marker of leukoplakia malignancy, while how changes in apolipoprotein B contribute to oral leukoplakia still requires further study.

## Data Availability

The original contributions presented in the study are included in the article/**Supplementary Material**. Further inquiries can be directed to the corresponding author.
